# Flying on their own wings: young and adult cuckoos respond similarly to long-distance displacement during migration

**DOI:** 10.1038/s41598-020-64230-x

**Published:** 2020-05-07

**Authors:** Kasper Thorup, Marta Lomas Vega, Katherine Rachel Scotchburn Snell, Regina Lubkovskaia, Mikkel Willemoes, Sissel Sjöberg, Leonid V. Sokolov, Victor Bulyuk

**Affiliations:** 1 Center for Macroecology, Evolution and Climate, Globe Institute, University of Copenhagen, Copenhagen, Denmark; 2 Saint Petersburg University, St Petersburg, Russia; 3 Biological Station Rybachy, Zoological Institute of Russian Academy of Sciences, St Petersburg, Russia

**Keywords:** Animal migration, Behavioural ecology

## Abstract

Common cuckoos *Cuculus canorus* are obligate nest parasites yet young birds reach their distant, species-specific wintering grounds without being able to rely on guidance from experienced conspecifics – in fact they never meet their parents. Naïve marine animals use an inherited navigational map during migration but in inexperienced terrestrial animal migrants unequivocal evidence of navigation is lacking. We present satellite tracking data on common cuckoos experimentally displaced 1,800 km eastward from Rybachy to Kazan. After displacement, both young and adult travelled similarly towards the route of non-displaced control birds. The tracking data demonstrate the potential for young common cuckoos to return to the species-specific migration route after displacement, a response so far reported exclusively in experienced birds. Our results indicate that an inherited map allows first-time migrating cuckoos to locate suitable wintering grounds. This is in contrast to previous studies of solitary terrestrial bird migrants but similar to that reported from the marine environment.

## Introduction

For a lone young common cuckoo *Cuculus canorus*, raised by foster-parents of other species, finding the proper wintering grounds thousands of kilometres away without conspecific guidance appears almost impossible. Nevertheless, as more than a billion other inexperienced migrants from the Palearctic do, it reaches its African wintering grounds every year^1,2^. How it manages to do this remains one of the most fascinating mysteries of migration biology^3^. Such challenges are faced across land and sea by a diverse range of organisms, including insects^4^ and cetaceans^5^. In the marine environment, inexperienced young of several species that travel independent of adults, including turtles^6^, salmon^7^ and eels^8^, are shown to rely on an inherited navigational map based on geomagnetic information^9^. This has the obvious advantage of being able to correct for unwanted displacement. Nevertheless, evidence for true navigation in first-time avian migrants remains equivocal^3^.

Routes and non-breeding staging sites of many long-distance migrating land birds are population-specific and conserved across individuals^10,11^ (but see^12^), with routes involving challenging barrier crossings such as the Mediterranean and the Sahara for Afro-Palearctic migrants. In general, young migrants possess an innate migratory orientation programme enabling them to overcome these challenges; a programme which over generations has provided the highest chances of returning to breed next spring and thus the highest fitness^13^. Many aspects of the adaptive programme have been revealed, such as innate timing and direction^14,15^. However, the evidence about whether it involves some form of navigation remains contradictory^3^.

Studying responses to displacement allows us to distinguish between different types of underlying navigation strategies^16^. Displacement experiments, such as the paradigmatic experiment by Perdeck^17^ involving more than 11,000 European starlings *Sturnus vulgaris* and later repeated with *Zonothricia* sparrows^18,19^, have documented an endogenous directional programme steering inexperienced migrants in a certain direction. Cage studies have shown that the direction is followed over an innately controlled period of time, providing first-time migrants with direction and distance^15^. With experience, this programme develops into a goal-area navigation programme normally allowing the birds to pinpoint at least their breeding and winter grounds even from unfamiliar areas^15^.

Such an ability has been well documented in adult birds as shown by lesser black-backed gulls *Larus fuscus*^20^ and adult common cuckoos^21^ returning to the normal migration route after long-distance displacements of 1,080 and 2,500 km, respectively, though with marked inter-individual flexibility in route and timing. Similar navigation responses have been shown for directional preferences of displaced adults^22^ but not always^23^. However, while the displacements of starlings and *Zonothricia* sparrows did not indicate such an ability in juveniles, contradicting evidence has been reported in other studies, and whether some ability to correct for orientation errors or wind displacement is also present in juvenile birds remains unclear^3^. A re-analysis of 109 published displacement experiments indicated that even juvenile birds were able to detect and react to displacements, including those in which displacement was simulated in a planetarium^24^. Additionally, experiments by Åkesson *et al*.^25^ indicate that juvenile white-crowned sparrows *Zonothricia leucophrys* may be able to correct for displacements and both wind-displaced and experimentally displaced birds on the Faroe Islands showed compensatory responses^26^.

**Figure 1:**
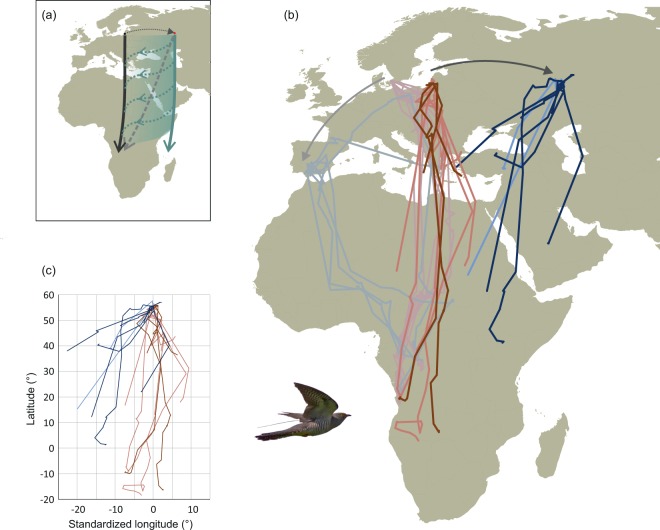
Tracks of control common cuckoos migrating through Rybachy, Kaliningrad, Russia, during autumn (red) and experimental birds displaced to Kazan, Russia (blue). (**a**) Standard uncompensated migration from Rybachy to the winter grounds (black), theoretical compensated route with navigation from the release site in Kazan to the normal winter grounds (grey dotted) and expected displacement responses (green) showing an uncompensated route (solid line) and adult-type response (dotted and transparent). (**b**) Tracks of adult (pale) and first time (dark) migrating cuckoos released in Rybachy (controls, red) and displaced (blue) to Kazan. Tracks of adults released in Denmark (pink) and displaced to Spain (grey) (Adapted from Willemoes *et al*.^21^ with permission) are shown for comparison. (**c**) Comparison of control and displaced tracks with standardised starting longitude. Maps are created using R software (version 3.4.4; https://cran.r-project.org/) using the packages “maps” version 3.3.0 and “rgdal” version 1.3-6.

Given the contradicting evidence about the ability to compensate displacement, we challenged this experimentally by building on recent advances in tracking technologies that allow globally unbiased spatiotemporal mapping of movements of smaller, long-distance migrants. Thus, we tracked the responses to an experimental 1,800 km eastward displacement of adult and young common cuckoos. Common cuckoos migrate from their breeding grounds in the Palearctic to their wintering grounds in sub-Saharan Africa. The long distances are travelled at night, presumably solitarily without guidance from conspecifics^1,2^. As the cuckoo is a nest parasite, juveniles rely on their inherent migration programme to locate suitable winter grounds^1,2,27^. We investigated the nature of the innate migration programme by comparing the tracks of displaced birds with those from non-displaced migrants caught *en route* on the Courish Spit at the Baltic Coast (Fig. 1a). We tested for evidence of navigation in young birds, potentially shown by displaced first-time migrants differing from non-displaced controls and responding similarly to adults compensating the displacement.

## Results

**Figure 2:**
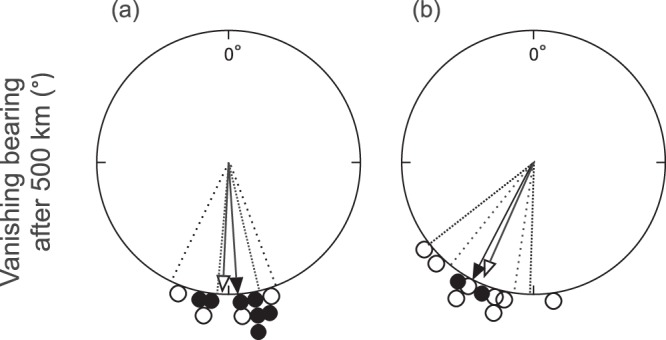
Directional response to displacement in first-time and adult cuckoos. Bearing after crossing 500 km in first-time (white) and adult (black) cuckoos from (**a**) Rybachy, Russia, (182.5 ± 14.9° and 175.7 ± 9.7° [mean ± s.d.] for juveniles (n = 4) and adults (n = 7), respectively) and (**b**) after the 1,800 km displacement eastward to Kazan, Russia, (203.5 ± 17.7° and 207.0 ± 5.7° for juveniles (n = 8) and adults (n = 2), respectively). Arrows indicate mean direction and vector length, dotted lines 95% CI.

**Figure 3:**
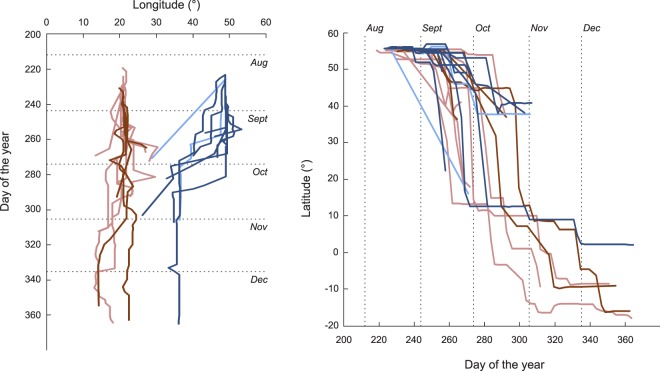
Timing of flights in displaced (blue) and control (red) adult (light) and young (dark) cuckoos. Displaced and control young were tracked for equally long (day of the year of last position: 289.3 ± 35.9 and 319.0 ± 47.7 [mean ± SD] for displaced (n = 8) and control young (n = 4), respectively; t_10_ = 1.22, p = 0.25; Day of the year of last position adults: 288.5 ± 24.7 and 307.3 ± 37.3 for displaced and control adults, respectively). The tracks were terminated for four out of the eight displaced juvenile cuckoos already in September and for the displaced adults in the end of September and early October.

The routes of first-time migrating cuckoos displaced eastwards from Rybachy to Kazan (Fig. 1b) led them overall closer to the normal migration routes, with the endpoints of the displaced first-time migrants being significantly displaced westward compared to the controls (−10.7 ± 7.1° and 0.0 ± 5.9° [mean ± s.d.] for displaced (n = 8) and control (n = 4) young, respectively; t-test of difference: p = 0.027; Fig. 1c. Bearings to endpoints of control young: 178.5°, r = 0.992, Rayleigh test: p = 0.008; displaced young: 202.7°, r = 0.976, Rayleigh test: p < 0.001; Watson-Williams test of difference, F_1,10_ = 10.708, p = 0.008). The displaced young cuckoos travelled south-southwest from Kazan in a slightly more westward direction than the control first-time migrants but their ability to correct only became apparent after they had travelled over a long distance, indicated by the initial bearings (after crossing 500 km), which were not significantly different (Fig. 2a,b). No differences in the timing of control and displaced birds were apparent (Fig. 3. Difference in timing of crossing 500 km distance: p = 0.128, 1000 km distance: p = 0.865; t-tests). Movements of young birds were on average slightly earlier – 10 days when crossing 500 km distance – but not significantly so and not after crossing 1,000 km (Difference in timing of crossing 500 km distance: p = 0.063, 1,000 km distance: p = 0.834; t-tests. Fig. 3).

**Figure 4:**
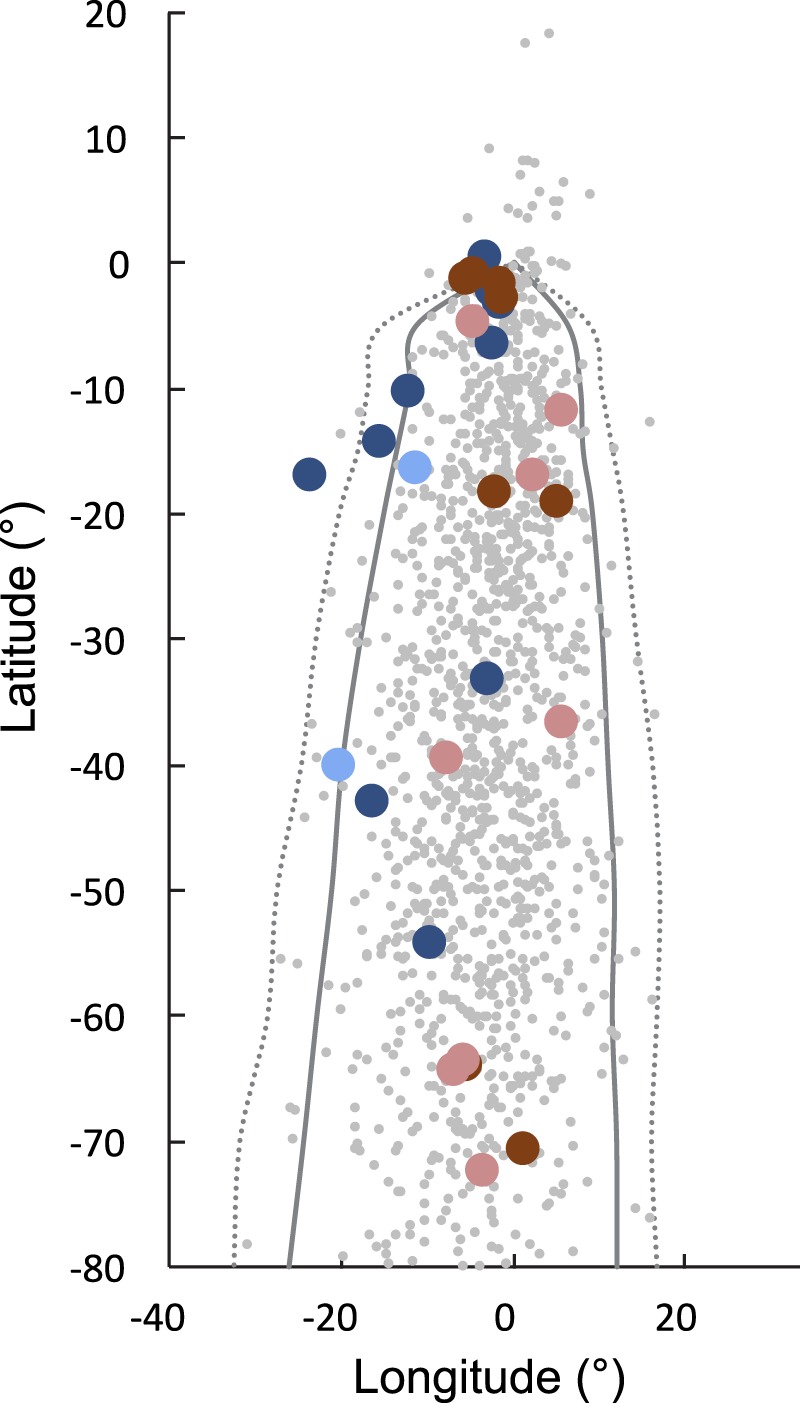
Comparison of simulated (grey) and observed endpoints of displaced (blue) and control (red) adult (light) and young (dark) cuckoos displaced to starting in (0°, 0°). Confidence intervals of simulated endpoints (dots; n = 1,500) are indicated (solid line: p < 0.05; stippled line: p < 0.001).

Though we find significant compensation in the displaced young on average, the individual responses varied and the resulting tracks showed a large scatter in directions and a large longitudinal spread along the migration route. Two birds demonstrated significant compensation (Outliers in simulation tests, p < 0.05 and p < 0.001, respectively; Fig. 4) both being more westerly than those of control birds with one individual reaching the longitudes of control birds and thus evidently compensating fully for the displacement. The tracks of displaced adults overlapped those of the displaced young with similar distances and degree of compensation (Fig. 1b,c) as did the directions to positions after crossing 500 km (Fig. 2b), and so the compensatory reactions among displaced young were similar to the compensation in displaced adults. Among the first-time migrants that compensated only partly (Figs. 1b,c and 2b), a bird wintering 2,500 km northeast of the nearest control bird demonstrates the potential for surviving in new wintering grounds without fully compensating for the displacement. This bird in fact moved toward south-southwest in January and February after having reached sub-Saharan Africa in October.

## Discussion

Our results provide new insights into the migratory programme that brings naïve parasitic cuckoos to their wintering grounds for the first time. Our tracks of displaced young demonstrate that it is possible for first time migrants to compensate for such a displacement and thus the existence of inherited navigational responses similar to those demonstrated in marine animals^6–8^. At the same time, other individuals followed routes with little or no compensation, although this may have changed if the birds were tracked all the way to the wintering grounds. The responses observed are similar to those observed in adults, which are also able to compensate for displacements but many nevertheless follow uncompensated routes long after the displacement. Overall, the tracks provide evidence of an adult-type response to long-distance displacement in juvenile common cuckoos.

Despite the evidence from animals navigating in the marine environment^6–8^, the capability of compensation after displacement in migratory naïve birds has not been unequivocally demonstrated before. Such an “open and flexible” system – similar to that found in adults – likely provides evolutionary advantages as birds have the “option” to stay if they find suitable routes and wintering areas but also the possibility of returning to the normal route. Compensatory responses might be needed less often for migrants in the terrestrial than in the marine environment as terrestrial migrants spend most of the time resting on the ground whereas marine animals are constantly in a flowing media^28^. Thus, compensation is typically only necessary for terrestrial migrants during travel when they are more exposed to for example winds. In fact, compensating partly would not need to be based on an inherited map: juveniles could combine orientation in the normal migration direction with experienced-based navigation toward a site already visited on migration, as birds already on the way on their first migration could be acquiring information necessary for later navigation^26^.

While individual birds’ responses to displacements are key for distinguishing navigation strategies^16^, most studies only track these in the lab^23,29^ or for a shorter distance (up to a few hundred kilometres)^26,30,31^. For long-distance migrants, satellite tracking allows spatially unbiased study of the behavioural responses to displacement but few experiments have been performed in first-time migrants, and all of these have focussed on social migrants where group orientation might be guided by experienced individuals. Displacement experiments with young white storks *Ciconia ciconia*^32^ and relocations of lesser spotted eagles *Clanga pomarina* for re-introduction^33^ indicated a less precise migration programme with birds being dependent on social interactions to complete normal migrations as also indicated by a study on hybrids between lesser and greater spotted eagles *Clanga clanga*^34^. The initial uncompensated movements could indicate navigation based on inherited “signposts”^35,36^ and they align well with observations from the marine environment, where navigation in inexperienced animals appears to be based on navigational markers (a simple look-up table^37^). Thus, the information might only be available when the animal is within a certain range, requiring long-distance tracking to observe.

An alternative explanation for the observed changes in routes by first-time migrating cuckoos would be that they travelled with experienced individuals. However, we believe this is unlikely to be the case because young cuckoos are mainly solitary migrants managing to reach their wintering grounds without guidance as shown by differences in timing between young and adults from other populations with other routes^2^. Nevertheless, the routes for populations naturally occurring at the site of displacement may well be similar to the displaced birds but flocking and social interactions such as calling are not regularly observed during migration. The overall navigation strategies employed by displaced birds appear similar between experienced and first-time migrants. The fact that many tracks ended without full compensation might be caused by transmission ending before having reached the wintering grounds. The less than full compensation in those ending far to the south could also be caused by a compensatory response rooted in a plane rather than a sphere. On a sphere, a certain difference in longitudes corresponds to a longer distance closer to the Equator and the distance between the capture and displacement sites is shorter than the distance between the normal wintering grounds and those that would be reached after uncompensated migration in displaced individuals. Thus, if the birds compensated the displacement distance (rather than a difference in longitudes) after having moved south they would not reach the longitudes of the control birds.

The actual mechanisms used for the navigational tasks remain unclear. Geomagnetic cues are widely available across taxa for orientation even in insects^38^. Given the omnipresence of geomagnetic cues and the demonstration of their use for true navigation in adult bird migrants^39,40^ as well as in inexperienced marine animals^6–9^ these appear to be the most likely candidates. Furthermore, experiments have indicated that geomagnetic cues, characteristic of certain latitudes or regions, affect the orientation^35^ or the deposition of fuel^36^ of first-time migrants. However, their use over inter-continental scales are not straightforward as neither field intensity, inclination nor declination show simple gradient patterns over the scales considered. Alternatively, navigation could be based on olfactory cues as in experienced migrating gulls^20^ and catbirds *Dumetella carolinensis*^31^.

Our results challenge the common understanding of the orientation programme in inexperienced migratory birds. The strategy appears to include some form of navigation, at least in common cuckoos. A navigational programme as demonstrated here might be crucial during even more extreme land bird migrations as for example the 13,000 km long migration spanning across many degrees of latitudes and longitudes of willow warblers *Phylloscopus trochilus* migrating from Chukotka to Southeast Africa^41^, but navigational responses at the scale considered has not been studied so far. Our study lays out the foundation for studying capabilities and underlying cue use necessary for a deeper understanding of bird migration. Broadening out our study with respect to populations and species awaits even further technical advancements such as those promised by ICARUS^42^.

## Methods

### Ethical statement

The Ethical Committee of the Zoological Institute (RAS #2015-01-14) and Kaliningrad Regional Agency for Protection, Reproduction and Use of Animal World and Forests approved sampling procedures and experimental manipulations, and permitted the catching and tagging of cuckoos. This study was carried out in accordance with Guidelines to the use of wild birds in research of the Ornithological Council^43^.The common cuckoo is not a protected species and it is not considered threatened or endangered.

### Experimental animals, tagging and displacement

Adult and juvenile cuckoos used for the study were caught in the Kaliningrad Region shortly after initiation of autumn migration during late July – August 2015-2018. Individual migration routes were tracked using satellite telemetry and individuals were either controls (tagged and released close to trapping site) or displaced (tagged and displaced in groups 1,800 km eastward to Kazan, Tatarstan).

Cuckoos were trapped at two sites: the Biological Station Rybachy (55° 9’ 13” N, 20° 51’ 29” E) and the Fringilla Field Station (55° 5’ 18” N, 20° 44’ 3” E). Two large Heligoland traps at the Fringilla station as well as mist-nests at the two stations were used for trapping. Playback of cuckoo song and calls was widely used to attract birds.

To treat both control and experimental birds equally, all birds were kept in aviaries after trapping for 1 to 22 days. They were provisioned with mealworms and water ad libitum until tagging and release. During this time, the birds’ behaviour and weight changes were monitored. Birds with high body mass (minimum >100 g) and good condition were tagged and released, or kept for planned displacement events if they had not been kept in captivity for too long (> 1 week). Control birds were released during the period both before and after displacements.

We fitted satellite transmitters on 27 juvenile and 18 adult common cuckoos (see Supplementary Table S1-2 for deployment details). Tags were fitted with a backpack harness of 2 mm ø braided nylon cord. The juvenile harness size was the same as the one used in adults to account for further body growth^44^. Cuckoos were tagged just before release (control birds), or one or two days before (displaced birds).

The displaced group consisted of 15 juveniles and 7 adults translocated to Kazan in 2015, 2016 and 2018. They were transported by commercial airline in pet carriers and released into a natural, wooded habitat in daylight conditions as soon as possible after arrival (release site 55° 51’ 18” N, 48° 48’ 18” E and 55° 39’ 22” N, 49° 2’ 13” E, respectively). The control group consisted of 12 juveniles and 11 adults. Apart from the early movement of two juveniles displaced to Kazan that moved more than 100 km within four days, we found no general difference between control and displaced birds in departure timing after release (Difference in days after first position when crossing 500 km distance: p = 0.154, 1,000 km distance: p = 0.920; t-tests).

### Tracking

The tags used for this study transmitted location information via satellites. Two models of transmitting tags were used to obtain spatial data in this study: Platform Terminal Transmitters (PTTs) 5 g solar PTT-100s (Microwave Telemetry Inc., Maryland, USA) and 3.4 g or 3.7 g PinPoint GPS ARGOS Tags 75 (LOTEK, Newmarket, Canada; tag mass depended on antenna specifications).

Location estimates were obtained from PTTs by interpreting the Doppler effect of transmissions from the tags to the Argos Satellite system. Position quality and accuracy is determined (where possible) by the number and duration of transmissions (estimated error classes 0: >1,500 m; 1: 500–1,500 m; 2: 250–500 m; 3: <250 m (^45^). The tags had a solar cell and the temporal schedule was 10 hours transmission followed by 48 hours off, for as long there was sufficient power.

The PinPoint tags provide GPS positions acquired at scheduled intervals, compressed and transmitted via the Argos system. The tags are scheduled in advance and limited by internal battery capacity (determined by the GPS acquisition time and transmission of data to the Argos system). In 2015, tags were programmed to obtain positions regularly on a short schedule and transmit data on a predefined date (estimable to a date prior to the Sahara crossing), or for a long schedule where positions were attempted until transmission which was determined by a critical battery voltage. In 2017, tags were equipped with Argos satellite pass prediction software by which the tag only transmitted data when a satellite was within range, thus substantially improving power efficiency and enabling regular transmission of data throughout the migration period. All transmission data include time stamps and satellite ID.

From the PinPoint tags, GPS position data were transmitted via the ARGOS satellite system and processed with LOTEK software; failed positions were excluded. Doppler positions can also be obtained from transmitting PinPoint tags but only a few of these were received.

In cases where an Argos transmission occurred but no Doppler position could be estimated, an estimate and error radii was calculated from satellite pass data by CLS. Where a PinPoint tag had acquired a concurrent GPS position this was used in preference to the ARGOS estimate.

After rejecting class Z estimates, we used the highest quality position from each transmission cycle closest to noon.

### Data analyses

Of the 27 tagged juveniles, we considered the 12 individuals that were tracked for more than 500 km/crossed south of 50°N to have started migration (Table 1). Initial movements appeared to be slightly further westward in the first year but the bearings after crossing 100 km were not significantly different from the other years, and thus in the analyses we considered results from all years together. In adults, 9 of 18 started migration.

**Table 1:** Summary of common cuckoo data. Numbers indicate individuals included in analyses, i.e. tracked for more than 500 km/crossing south of 50°N.

Year	Juveniles		Adults		Total
	Rybachy	Kazan	Rybachy	Kazan	
2015	1 (5)	3 (5)	3 (6)	1 (5)	8 (21)
2016	1 (2)	1 (5)			2 (7)
2017	2 (5)		4 (5)		6 (10)
2018		4 (5)		1 (2)	5 (7)
All years	4 (12)	8 (15)	7 (11)	2 (7)	21 (45)

Numbers in brackets indicate numbers of individuals tagged.

We used the highest quality position for every duty cycle obtained from ARGOS/CLS^44^. Several birds moved back and forth for some time after release with no consistent direction of movement, and we considered that they started migration when consistent directions were observed.

We investigated the spatiotemporal pattern of responses by grouping individuals’ locations according to when they had passed certain distances. However, the sampling schedule did not allow detailed analyses of timing of responses. We compared bearings and timings after crossing 500 km (n = 13) and 1,000 km (n = 19) from the release areas because for shorter intervals there were not enough locations for meaningful comparisons (only up to a maximum of 7 in 100 km intervals). The directedness of sample orientations for the individuals of a group was tested with the Rayleigh test. Differences in sample orientations were tested with Watson-Williams tests. We used t-tests on day of the year of crossing 500 km and 1,000 km as well as reaching endpoints to compare the timing of migration between controls and displaced and between adult and juveniles.

Because of the distance to the wintering grounds, even large distances at the latitude of the wintering grounds result in potentially relatively small differences in bearings. Thus, in addition to comparing bearings to endpoints (last position, see Supplementary Table S2 for details) using a Watson-Williams test, we tested for evidence of navigation in young birds by comparing the westward longitudinal displacement of endpoints in displaced versus controls. We treated longitude data as linear and compared groups with a t-test. This was justified as less than 10% of the full circle of longitudes were traversed and the Watson-Williams test based on longitudes reported the same result (F_1,10_ = 6.701, p = 0.027 for a difference between controls and displaced). In an ANOVA including both age and displacement effects together, displacement was significant but age was not (p = 0.07) potentially because of small sample size.

To test for displaced individuals compensating for the displacements, we compared with tracks simulated under the assumption that control birds follow a clock-and-compass strategy (vector orientation). Outliers (i.e. significantly deviating longitudinally), were considered compensating if the deviation was in the direction of compensation (Fig. 4). In the young cuckoos, we identified 37 movements longer than 100 km that were used for simulations. Considering the longitudes and latitudes traversed in each of these as the result of a clock-and-compass step, we simulated tracks by adding from 1 to 15 randomly chosen longitudes/latidues from this set. For each total number of steps (1 to 15), we simulated 1,000 tracks, resulting in a total of 15,000 simulated endpoints for the test data.

We used R 3.3.2^46^ for linear statistics and Oriana 3.0^47^ for circular statistics and diagrams. MS Excel was used for simulations.

## Supplementary information

Supplementary Information.

## Data Availability

All tracking data are available in www.movebank.org (10.5441/001/1.vk36vq82)
