# Supplementary material for: Flying on their own wings: young and adult cuckoos respond similarly to long-distance displacement during migration
Source: Sci Rep. 2020 May 7;10:7698. doi: 10.1038/s41598-020-64230-x (PMC7205979; doi:10.1038/s41598-020-64230-x)
Supplement: Supplementary file 1 — Supplementary Information. [file 41598_2020_64230_MOESM1_ESM.docx]

Supplementary Information

Flying on their own wings: young and adult cuckoos respond similarly to long-distance displacement during migration

Kasper Thorup, Marta Lomas Vega, Katherine Rachel Scotchburn Snell, Regina Lubkovskaia, Mikkel Willemoes, Sissel Sjöberg, Leonid V. Sokolov, Victor Bulyuk

This PDF files include:

Supplementary Table S1: Tag deployment details of all tagged cuckoos

Supplementary Table S2: Information on release site, date of first position, tag type and validation of last position for each individual included in the analyses

**Table S1. Tag deployment details of all tagged cuckoos.** Tag types are indicated as number of PTTs (5 g solar PTT-100s) plus number of PP (3.4 g [in 2015 only] or 3.7 g PinPoint GPS Argos). Numbers indicate individuals included in the analyses. Numbers in brackets indicate numbers of individuals tagged.

|  | Juveniles |  |  |  |  | Adults |  |  |  |  | All ages |  |
| --- | --- | --- | --- | --- | --- | --- | --- | --- | --- | --- | --- | --- |
| Year | Rybachy |  | Kazan |  |  | Rybachy |  | Kazan |  |  | Total |  |
|  | PTT | PP | PTT | PP |  | PTT | PP | PTT | PP |  | PTT | PP |
| 2015 | 1 (5) |  | 3 (5) |  |  | 1 (1) | 2 (5) |  | 1 (5) |  | 5 (11) | 3 (10) |
| 2016 | 1 (2) |  | 1 (5) |  |  |  |  |  |  |  | 2 (7) |  |
| 2017 |  | 2 (5) |  |  |  | 4 (5) |  |  |  |  | 4 (5) | 2 (5) |
| 2018 |  |  | 2 (2) | 2 (3) |  |  |  | 1 (2) |  |  | 3 (4) | 2 (3) |
| All years | 2 (7) | 2 (5) | 6 (12) | 2 (3) |  | 5 (6) | 2 (5) | 1 (2) | 1 (5) |  | 14 (27) | 7 (18) |

**Table S2. Information on release site, date of first position, tag type and validation of last position for each individual included in the analyses.**

| Argos ID | Age | Release site | First position | Tag type | First day of last position | Validation of last position |
| --- | --- | --- | --- | --- | --- | --- |
| 148415 | Adult | Rybachy | 2015-08-07 | PinPoint GPS | 2015-09-29 | No movements longer than 0.01 km from this site, last transmission 2015-12-02 |
| 151035 | Adult | Rybachy | 2015-08-12 | PinPoint GPS | 2015-09-26 | No movements longer than 0.2 km from this site, last transmission 2015-11-21 |
| 151033 | Adult | Kazan | 2015-08-12 | Satellite pass prediction | 2015-09-28 | Last transmission |
| 135533 | Adult | Rybachy | 2015-09-07 | PTT-100 | 2015-12-30 | Last transmission of the year, last transmission 2017-03-02 |
| 168807 | Adult | Rybachy | 2017-08-17 | PTT-100 | 2017-10-19 | Last transmission |
| 168810 | Adult | Rybachy | 2017-08-17 | PTT-100 | 2017-12-17 | Last transmission of the year, last transmission 2018-01-20 |
| 168806 | Adult | Rybachy | 2017-08-18 | PTT-100 | 2017-11-08 | No movement longer than 3.3 km from this site, last transmission 2017-11-17 |
| 168808 | Adult | Rybachy | 2017-08-18 | PTT-100 | 2017-10-18 | Last transmission |
| 168734 | Adult | Kazan | 2018-09-02 | PTT-100 | 2018-11-02 | Last transmission |
| 150964 | Juvenile | Kazan | 2015-08-11 | PTT-100 | 2015-12-31 | Last transmission of the year, last transmission 2016-08-27 |
| 150968 | Juvenile | Kazan | 2015-08-11 | PTT-100 | 2015-09-30 | Last transmission of the year, last transmission 2018-10-18 (with no movements after 2015) |
| 150967 | Juvenile | Kazan | 2015-08-12 | PTT-100 | 2015-09-13 | Transmission furthest from release site (distance from start position 771 km), transmission afterwards closer to release site, last transmission 2015-09-29 |
| 150966 | Juvenile | Rybachy | 2015-08-19 | PTT-100 | 2015-12-29 | Last transmission of the year, last transmission 2016-03-07 |
| 157309 | Juvenile | Kazan | 2016-08-22 | PTT-100 | 2016-09-14 | No movements longer than 3 km from this site, last transmission 2018-12-18 |
| 157303 | Juvenile | Rybachy | 2016-08-23 | PTT-100 | 2016-09-21 | No movements longer than 4.1 km from this site, last transmission 2017-12-12 |
| 40552 | Juvenile | Rybachy | 2017-08-27 | PinPoint GPS | 2017-10-20 | Last transmission |
| 40536 | Juvenile | Rybachy | 2017-09-02 | PinPoint GPS | 2017-12-21 | Last transmission of the year, last transmission 2018-01-10 |
| 61851 | Juvenile | Kazan | 2018-08-28 | PinPoint GPS | 2018-10-30 | No movements longer than 0.01 km from this site, last transmission 2018-11-27 |
| 61860 | Juvenile | Kazan | 2018-08-28 | PinPoint GPS | 2018-10-09 | Last transmission |
| 168733 | Juvenile | Kazan | 2018-09-02 | PTT-100 | 2018-09-27 | Last transmission |
| 168735 | Juvenile | Kazan | 2018-09-03 | PTT-100 | 2018-11-03 | No movements longer than 0.6 km from this site, last transmission 2018-11-08 |
